# Supplementary material for: Nutrition-Related N-of-1 Studies Warrant Further Research to Provide Evidence for Dietitians to Practice Personalized (Precision) Medical Nutrition Therapy: A Systematic Review
Source: Nutrients. 2023 Apr 4;15(7):1756. doi: 10.3390/nu15071756 (PMC10097352; doi:10.3390/nu15071756)
Supplement: Supplementary file 1 [file nutrients-15-01756-s001.zip › nutrients-2276463-supplementary.pdf]

Supplementary Table S1 Search strategies employed to locate the N-of-1 studies

Scopus search

TITLE-ABS-KEY ((n-of-1 OR n-of-one OR single-patient OR single-subject OR individual-patient) AND (diet* OR food* OR snack* OR beverage* OR personalised AND nutrition* OR personalized AND nutrition* OR precision AND nutrition* OR eating AND pattern* OR meal AND pattern* OR protein* OR carbohydrate* OR sugar* OR macronutrient OR micronutrient OR energy OR calori* OR intake* OR consumption OR nutri* OR fat OR fats OR eat* OR feed OR feeds OR feeding*)) AND (LIMIT-TO (PUBYEAR , 2022) OR LIMIT-TO (PUBYEAR , 2021) OR LIMIT-TO (PUBYEAR , 2020) OR LIMIT-TO (PUBYEAR , 2019) OR LIMIT-TO (PUBYEAR , 2018) OR LIMIT-TO (PUBYEAR , 2017) OR LIMIT-TO (PUBYEAR , 2016) OR LIMIT-TO (PUBYEAR , 2015) OR LIMIT-TO (PUBYEAR , 2014) OR LIMIT-TO (PUBYEAR , 2013) OR LIMIT-TO (PUBYEAR , 2011) OR LIMIT-TO (PUBYEAR , 2010) OR LIMIT-TO (PUBYEAR , 2009) OR LIMIT-TO (PUBYEAR , 2008) OR LIMIT-TO (PUBYEAR , 2007) OR LIMIT-TO (PUBYEAR , 2006) OR LIMIT-TO (PUBYEAR , 2005) OR LIMIT-TO (PUBYEAR , 2003) OR LIMIT-TO (PUBYEAR , 2001)) AND (EXCLUDE (PREFNAMEAUID , "Więckowska-Rusek, K.#57572202700"))

Search Strategy used for Medline, Embase, Cochrane Central and PsychInfo

#	**Searches**
1	“N-of-1 trial*”.mp.
2	“N-of-1 method*”.mp.
3	“N-of-1 design*”.mp.
4	“Single-patient study”.mp.
5	“Single-patient studies”.mp.
6	“Single-patient trial*”.mp.
7	“Single-patient design*”.mp.
8	“Single-subject trial*”.mp.
9	“Single-subject study”.mp.
10	“Single-subject studies”.mp.
11	“Individual-patient trial*”.mp.
12	“Individual-patient studies”.mp.
13	“Individual-patient studies”.mp.
14	Diet*.mp.
15	Snacks/
16	Feeding Behavior/
17	Beverages/
18	Nutrition Therapy/
19	Personalised nutrition*.mp.
20	Personalized nutrition*.mp.
21	Precision nutrition*.mp.
22	Eating pattern*.mp.
23	Meal pattern*.mp.
24	Food/
25	Protein*.mp.
26	Carbohydrate*.mp.
27	Sugar*.mp.
28	Macronutrient*.mp.
29	Micronutrient*.mp.
30	Energy.mp.
31	Calori*.mp.
32	Intake*.mp.
33	Consumption.mp.
34	Nutri*.mp.

35	Fat.mp.
36	Fats.mp.
37	Eat*.mp.
38	Feed.mp.
39	Feeds.mp.
40	Feeding*.mp.
41	Single-Case Studies as Topic/
42	1 or 2 or 3 or 4 or 5 or 6 or 7 or 8 or 9 or 10 or 11 or 12 or 14 or 41
43	14 or 15 or 16 or 17 or 18 or 19 or 20 or 21 or 22 or 23 or 24 or 25 or 26 or 27 or 28 or 29 or 30 or 31 or 32 or 33 or 34 or 35 or 36 or 37 or 38 or 39 or 40
44	42 and 43
45	Limit 44 to (english language and humans and yr="2000-current")

Table S2. Quality of dietary assessment checklist developed for this review.

ALL METHODS	Scoring
Document dietary assessment method	i.e. diet recall, food frequency questionnaire (FFQ), or other dietary questions, ecological momentary assessment (EMA), (weighed) food record
Is it an appropriate tool to answer the study question and for use in the target population?	If NO → assign POOR quality of dietary assessment but continue questions<br>If YES → continue questions
Any referenced or reported validation study?	If yes, use to complete question 1a-b.
Document validation method	i.e. diet recall, food frequency questionnaire, (weighed) food record, biomarker (list), other (describe)
1a. Validation study sample & sample size (max 1 point):	0.5 if validated in same population as for intervention study; PLUS 0.5 if n≥100 or n≥50 if biomarkers used.
1b. Statistics to assess validity<br>(max 2 points)	0.5 if compare/test mean or median or difference or face validity (expert review); PLUS [highest value of: 0.25 correlation OR 0.5 adjusted correlations/ unweighted Kappa/ Cronbach alpha OR 0.75 deattenuated/ interclass correlations/ weighted Kappa]; PLUS 0.5 classification or Bland & Altman plot; PLUS 0.25 for use of more than one validation method
2. Data collection and analysis (max 1.5 points)	0.5 if researcher administered (i.e. supervised, face to face or phone interview); OR 0.5 if subjects trained for automated data collection such as recall and EMA; PLUS 0.5 if reviewed/checked by a trained person before analysis PLUS 0.5 if relevant nutrient databases reported
RECALL METHOD	
3a. Number of days recall (max 1 point)	0.5 for multiple days of recall: 0.5 for consideration of all days of the week (i.e. weekday/ weekend day)
3b. Use of multiple pass and aids/ prompts (max 0.5 points)	(0.25 if multiple pass protocol used: 0.25 if aids/ prompts used for portion size estimation)
FOOD RECORD	
3a. Number of days recorded (max 1 point)	0.5 for 4 or more days: 0.5 for consideration of all days of the week
3b. Use aids/ prompts (max 0.5 points)	(0.25 if aids/ prompts/ weight used for portion size estimation; 0.25 for coding/analysis by a trained person OR 0.25 a fully automated online method such as an app)
DIETARY QUESTIONNAIRE	

3a. Details provided (max 1 point)	1.0 point if questions reported OR 0.5 points if summary of items reported.
3b. Frequency (max 0.5 points)	0.25 points if interval covered by questions documented and 0.25 robust portion size estimation methodology
EMA METHODS	
3a (max 1 point)	0.5 if questions supplied; 0.25 for 4 or more days recording; 0.25 for consideration of weekend and weekdays;
3b. (max 0.5 point)	0.5 for reporting compliance with prompts;
**Total Score (max 6 points)**	**<3 points (poor); ≥3 and <5 points (acceptable); ≥5 points (good)**
